# Zinc Phthalocyanine Labelled Polyethylene Glycol: Preparation, Characterization, Interaction with Bovine Serum Albumin and Near Infrared Fluorescence Imaging *in Vivo*

**DOI:** 10.3390/molecules17096348

**Published:** 2012-05-25

**Authors:** Feng Lv, Bo Cao, Yanli Cui, Tianjun Liu

**Affiliations:** Tianjin Key Laboratory of Biomedical Materials, Institute of Biomedical Engineering, Chinese Academy of Medical Sciences & Peking Union Medical College, Tianjin 300192, China

**Keywords:** zinc phthalocyanine, polyethylene glycol, near infrared fluorescence imaging

## Abstract

Zinc phthalocyanine labelled polyethylene glycol was prepared to track and monitor the *in vivo* fate of polyethylene glycol. The chemical structures were characterized by nuclear magnetic resonance and infrared spectroscopy. Their light stability and fluorescence quantum yield were evaluated by UV-Visible and fluorescence spectroscopy methods. The interaction of zinc phthalocyanine labelled polyethylene glycol with bovine serum albumin was evaluated by fluorescence titration and isothermal titration calorimetry methods. Optical imaging *in vivo*, organ aggregation as well as distribution of fluorescence experiments for tracking polyethylene glycol were performed with zinc phthalocyanine labelled polyethylene glycol as fluorescent agent. Results show that zinc phthalocyanine labelled polyethylene glycol has good optical stability and high emission ability in the near infrared region. Imaging results demonstrate that zinc phthalocyanine labelled polyethylene glycol can track and monitor the *in vivo* process by near infrared fluorescence imaging, which implies its potential in biomaterials evaluation *in vivo* by a real-time noninvasive method.

## 1. Introduction

With the wide application of biomaterials, a critical point in the design of biomaterials is the safety, biocompatibility and degradability of biomaterials and whether there are any differences between the *in vivo* and *in vitro* mechanisms [1,2]. Traditionally, the most frequently applied analytical method is analysis via histology [3,4]. However, histology is an end point inherently destructive measurement and it excludes serial time studies on a single animal. The technique presents an obstacle to real-time studies in more complex environments *in vivo*. *In vivo* monitoring with noninvasive or microinvasive skill maybe develops as an appropriate technique to investigate the fate of biomaterials. Monitoring the fate of the drug carriers or implant biomaterials *in vivo* easily by noninvasive methods could beneﬁt the design of biomaterials [5,6]. Ultrasonography and magnetic resonance imaging have been applied to track and monitor the fate of biomaterials *in vivo*. Solorio reported that diagnostic ultrasound could be applied to visualize and quantify the process of implant formation *in vivo* [7]. Mader characterized drug release and polymer degradation *in vivo* by electron paramagnetic resonance and magnetic resonance imaging [8].These medical imaging methods have provided beneficial information of biomaterials *in vivo*, which advances real-time and objective evaluation of carriers or implant biomaterials.

Fluorescent optical imaging as a novel imaging modality could have more wider application in biomedical fields due to its advantages such as low cost, non-ionic low-energy radiation, high sensitivity, continuous real-time monitoring, and its noninvasive or minimally invasive nature [9,10]. Furthermore, near infrared fluorescence imaging can increase sensitivity and penetration depth in biological tissues and organs for detection and imaging, because bioorganisms have low scattering effects and background interference in the near infrared region [11,12]. Near infrared imaging methods have been applied in several medical fields including the diagnosis of cancer, vascular mapping, tissue perfusion, inﬂammation, atherosclerosis and protease activity [13,14,15,16]. Nowadays, fluorescence imaging has been exploited to track or monitor the fate of biomaterials [17,18,19,20]. Artzi *et al.* investigated *in vivo* and *in vitro* tracking of erosion in biodegradable hydrogels using fluorescein and Texas red. This approach enables rapid *in vitro* screening of materials by fluorescence imaging [17]. Lovell *et al.* demonstrated porphyrin cross linked hydrogels for monitoring and surgical resection [18]. Cunha-Reis *et al.* used tetramethylrhodamine isothiocyanate labelling of chitosan to monitor the degradation of chitosan for tissue engineering and identiﬁed the dispersion pathway of the chitosan membrane degradation products *in vivo* [19]. Moller *et al.* synthesized Lucifer yellow tagged hydrogels and monitored the *in vivo* process by fluorescence imaging [20]. These fluorescence tags have emissions below 650 nm. To enhance the sensitivity, novel fluorescence labels with long wavelength emission need to be developed.

Zinc phthalocyanines present intense ﬂuorescence in the near infrared region. This property has caused them to be widely investigated in materials sciences, photochemistry or biomedical sciences [21,22,23]. Based on the optical sensitivity of phthalocyanines, they were often applied as photodynamic therapy (PDT) agents to treat cancer or against bacteria [24,25,26,27,28,29]. Besides, zinc phthalocyanines were reported recently as optical probes either *in vitro* or *in vivo* due to the low absorbance of biosystems in the near infrared spectral window [30,31,32]. Nesterova *et al.* studied phthalocyanine dimerizationds as near infrared fluorescence probes for *in vivo* and *in vitro* DNA/RNA detection [30]. Mantareva *et al.* reported cationic zinc phthalocyanines as advanced fluorescent contrast agents for pigmented melanoma tumors [31]. We have demonstrated that zinc phthalocyanine as an optical imaging probe can show good imaging *in vivo* [32]. Zinc phthalocyanines maybe become novel labeling molecules to track the biomaterials *in vivo*.

Polyethylene glycol(PEG) as an important type of hydrophilic polymer has been often used in biomedical applications such as bio-conjugation, drug carriers and tissue engineering owing to itscritical properties including good biocompatibility, non-immunogenity and resistance to protein adsorption [33,34,35]. *In vivo* metabolism of PEG is a key point to the design and application of PEG derivatives as implants or carriers. Tracking polethylene glycol *in vivo* can investigate the distribution and metabolism in real-time. Thus the safety, biocompatibility and degradability of PEG are studied *in vivo* and analytical methods *in vitro* or *ex vivo* excluded. It is significant and helpful to elucidate the fate of PEG *in vivo* by imaging techniques. PEG hydrogel conjugated with porphyrin was monitored for surgical resection by fluorescence imaging [18]. Due to the limitations of the emission group, the depth of imaging and sensitivity maybe suffer some deficiencies. Zinc phthalocyanine labelled PEG can track and monitor the *in vivo* processes of PEG effectively because of its long wave length emission. PEG conjugated zinc phthalocyanine has been studied as a PDT agent because PEG can improve the solubility of zinc phthalocyanine [36,37]. The authors modified zinc phthalocyanine with four short PEG chains or one lateral functional PEG group. Each of them shows good biocompatibility and some PDT effect. In this work, zinc phthalocyanine was linked to PEG as a fluorescence label in order to interpret the fate of biomaterials *in vivo*. Zinc phthalocyanine labelled PEG was prepared and characterized. We found that the zinc phthalocyanine labelled PEG retained the ﬂuorescent property of zinc phthalocyanine and could track PEG *in vivo*. Combining zinc phthalocyanines with PEG would strengthen the applications of zinc phthalocyaninesin fluorescence molecular imaging for tracking biomaterials *in vivo*.

## 2. Results and Discussion

**Scheme 1:**
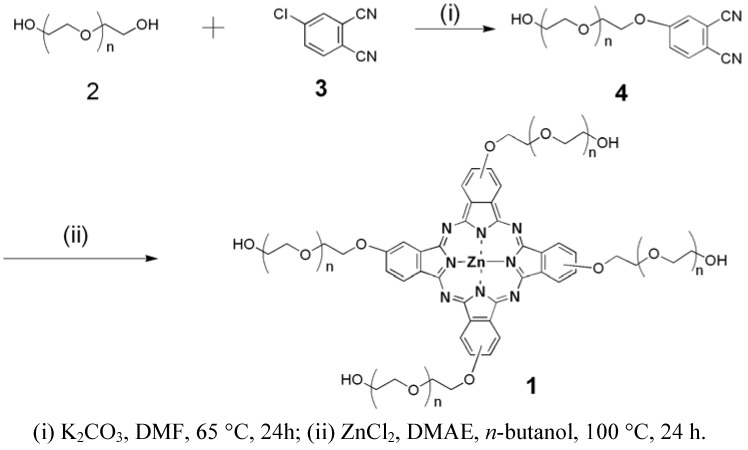
Synthetic route to zinc phthalocyanine labelled PEG.

**Figure 1:**
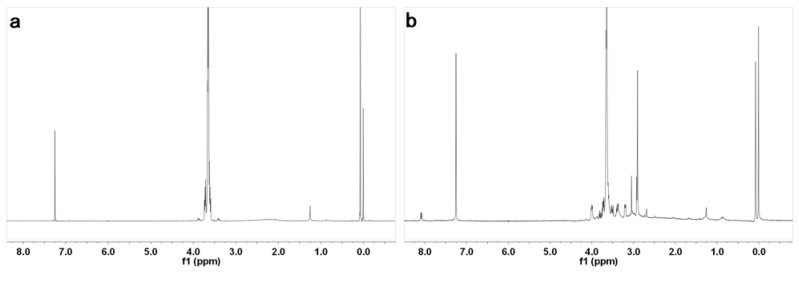
H-NMR of PEG (**a**) andzinc phthalocyanine labelled PEG (**b**).

**Figure 2:**
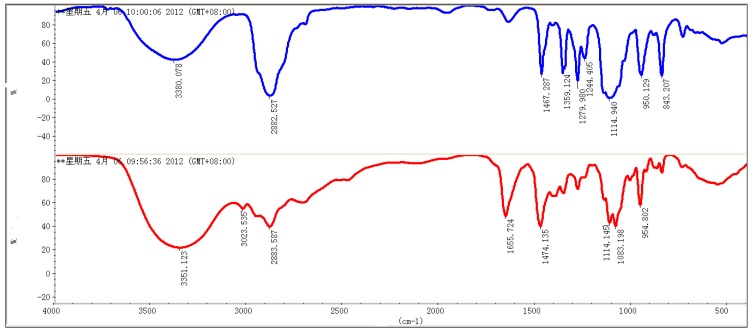
Infrared spectra of zinc phthalocyanine labelled PEG (red) and PEG (blue).

Zinc phthalocyanine labelled PEG was prepared using the two-step synthesis shown in Scheme 1. Zinc phthalocyanine conjugated PEG was usually prepared by condensation of PEG conjugated phthalonitrile [37]. In the synthesis of PEG conjugated, PEG was reacted with 4-chlorophthalonitrile instead of the commonly used 4-nitrophthalonitrile [37]. Compared to the traditional synthetic route using 4-nitrophthalonitrile, a higher yield was obtained by the substitution reaction of the hydroxyl compound with 4-chlorophthalonitrile. Substitution reactions are a common synthetic route for the water-soluble zinc phthalocyanines [38]. In our prior study, water-soluble glucose conjugated zinc phthalocyanines were prepared in high yield by this route [32]. In the condensation reaction of PEG conjugated phthalonitrile, amixedsolution of DMAE and *n*-butanol was used according to the preparation of glycoconjugated phthalocyanines [32,39]. The application of the mixed solution can enhance the yield [39] because DMAE dissolves both the starting reactant and the formed macro cycle well. The formed phthalocyanine in solution during the course of the template reaction partially leads to a decrease in yield. The addition of *n*-butanol allowed the formed phthalocyanine to precipitate from the solution and reduced this effect. Zinc phthalocyanine labelled PEG was purified by precipitation with cooled diethyl ether, which was usually applied to the purification of conjugated PEG as a simple and convenient method. The purification differs from the commonly method of zinc phthalocyanine short chain PEG purification due to the conjugation of the long chain PEG [40] The chemical structures were characterized by ^1^H-NMR and FT-IR spectroscopy. The ^1^H-NMR spectra of PEG and zinc phthalocyanine conjugated PEG are shown in Figure 1.The characteric peaks of PEG are the –OH peak at 1.5 ppm and –CH_2_ peak at 3.6 ppm. In the spectrum of zinc phthalocyanine labelled PEG, several sharp –CH_2_ peak sare seen from 2.7 ppm to 4.0 ppm due to the assymmetry of the one terminal group of zinc phthalocyanine in addtion to the characteric peak of –OH at 1.3 ppm in PEG. Additionally, a minor peak at 8.1 ppm is the signal of the Ar-H of phthalocyanine, which is not obvious due to the shielding from the long PEG chain. To verify the structure of zinc phthalocyanine labelled PEG, its FT-IR spectrum was recorded. As shown in Figure 2, characteristic peaks of zinc phthalocyanine conjugated PEG and PEG are seen. The peaks at 3380, 2982, 1467, 1141 cm^−1^ are the signals of OH, CH_2_ and C-O of PEG. In the spectrum of zinc phthalocyanine labelled PEG, additiaonal peaks are observed besides the characteristic peaks of PEG which are retained. The peaks of C=N at 1655 cm^−1^ and N-H at 3023 cm^−1^ are the characteristic signals of zinc phthalocyanine. All these signals illustrate zinc phthalocyanine has been successful attached to the PEG molecule.

**Figure 3:**
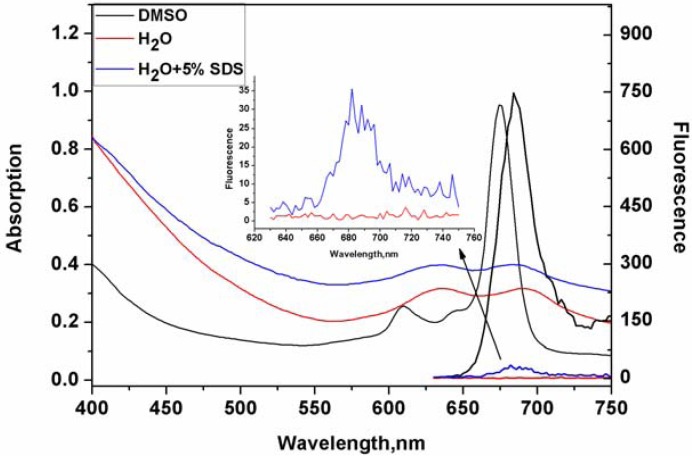
UV absorption and fluorescence spectra of zinc phthalocyanine labelled PEG in DMSO, H_2_O and 5%SDS solution at the concentration of 20 μM.

Zinc phthalocyanine conjugated PEG has beneficial water solubility owing to the hydrophilicity of the ethylene glycol group. Compared with other zinc phthalocyanine short chain PEGs, zinc phthalocyanine conjugated PEG 800 is more soluble in water solution because of the extension of the ethylene glycol monomer. Optical behaviors of zinc phthalocyanine labelled PEG in DMSO and water solution are shown in Figure 3. In DMSO solution, a typical absorption of an intense and sharp Q-band in the near-infrared area at 610 nm and 670 nm indicates the non-aggregated state of zinc phthalocyanine labelled PEG. Fluorescence emission is shown at 690 nm in DMSO with excitation at 610nm (Φf = 0.37). The major advantage of zinc phthalocyanines over porphyrins is that they have longer wavelength absorptions and much higher intensity than the Q bands of porphyrins. This demonstrates that zinc phthalocyanine is a better potential label for biomaterials than porphyrins although porphyrins have been applied to track biomaterials [18]. Just like other water soluble zinc phthalocyanines [28], zinc phthalocyanine labelled PEG also presents aggregation states in water with a standard M peak. The absorption peak in water is much lower and broader than in DMSO solution and the Stokes shifts in water are less than in DMSO solution by some 30 nm. In addition, little fluorescence signal is shown in water from the fluorescence spectrum. Additionally, the optical behavior of zinc phthalocyanine labelled PEG was investigated by using a surfactant like SDS at aconcentration of 5%. From the absorption and emissionspectrain SDS solution,the fluorescence intensity of zinc phthalocyanine labelled PEG greatly increasesin comparison to that in water although the absorption peak does not alter obviously. These results demonstrate that it cannot reach non-aggregation states like in DMSO solution, but some disaggregation happens with the help of surfactant. However,molecular aggregation in water solution does not limit imaging effect *in vivo* in the following experiments. The same phenomenon has been observed before in other water soluble phthalocyanines [28]. The fact that aggregation disappears maybe results from the presence of protein, so it is important and significant to investigate the interaction with proteins of phthalocyanine labelled PEG. Based on disaggregation in biological environment of complex biomolecules, zinc phthalocyanine labelled PEG can emit suitable fluorescence *in vivo*.

**Figure 4:**
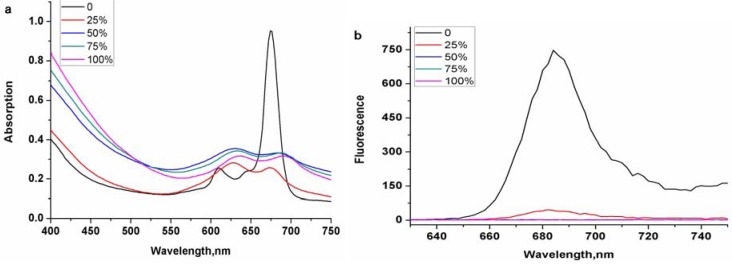
The aggregation behavior of zinc phthalocyanine labelled PEG by UV-Vis (**a**) and fluorescence spectra (**b**) using DMSO/water mixed solution with water ratio of0%, 25%, 50%, 75% and 100% at the concentration of 20 µM.

In order to further consider the solvent aggregation effect of zinc phthalocyanine labelled PEG, a series of mixed solutions of water and DMSO were chosen to measure their optical behavior (Figure 4). Zinc phthalocyanine labelled PEG in 75 vol.% DMSO shows less aggregation, but the aggregation behavior is seen obviously with the increase of water ratio in mixed sulution.

**Figure 5:**
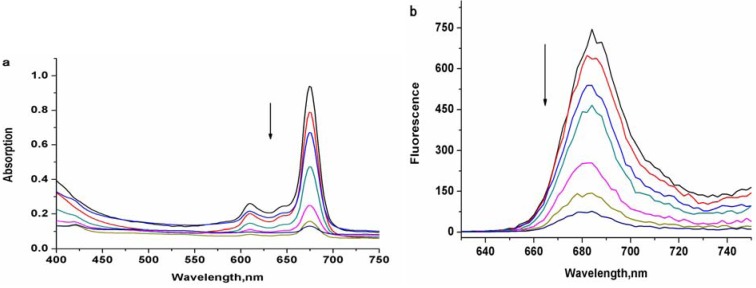
Absorption spectra (**a**) and fluorescence spectra (**b**) of zinc phthalocyanine labelled PEG in the concentration range from 1 µM to 20 µM (1, 2, 4, 8, 12, 16, 20 µM).

When measured in less than 50 vol.% DMSO, it shows serious aggregation with little fluorescence emission.Although other water soluble phthalocyanines have shown the same phenomenon, zinc phthalocyanine labelled PEG has more intensive aggregation owing to the long PEG chain. The concentration dependence of zinc phthalocyanine labelled PEG was investigated at concentrations ranging from 1 to 20 μM in DMSO solution (Figure 5). With the decrease of concentration, the absorption and emission degrade accordingly with a linear relationship. Optical signal is shown obviously even only at low concentration of 1 μM. These results confirm the sufficient stability and beneficial emmission of zinc phthalocyanine labelled PEG. 

**Figure 6:**
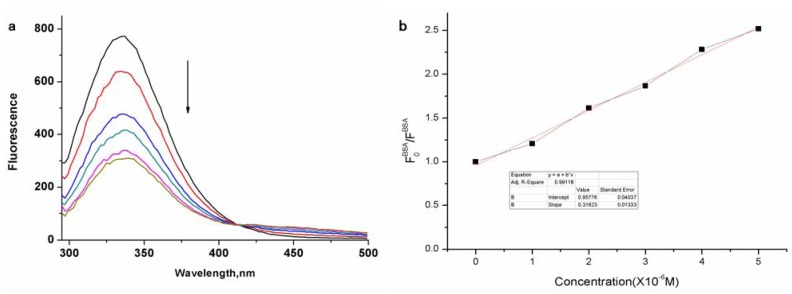
Fluorescence emission spectral changes of BSA (**a**) and linear relationship (**b**) on the addition of varying concentrations of zinc phthalocyanine labelled PEG and PEG in water (0, 1, 2, 3, 4, 5 μM).

Drugs, carriers or biomaterials interactions with plasma proteins are of considerable pharmacological importance because the effects on organs or tissues depends on their binding to plasma proteins. The high affinity to albumin will hinder *in vivo* effects. Bovine serum albumin (BSA) in aqueous media is usually used to investigate the interaction as a protein model. Fluorescence quenching method and isothermal titration calorimetry (ITC) measurements can analyse the interation of zinc phthalocyanine labelled PEG with BSA qualitatively and quantitately. The fluorescence spectra of zinc phthalocyanine labelled PEG titrated BSA are shown in Figure 6.

**Figure 7:**
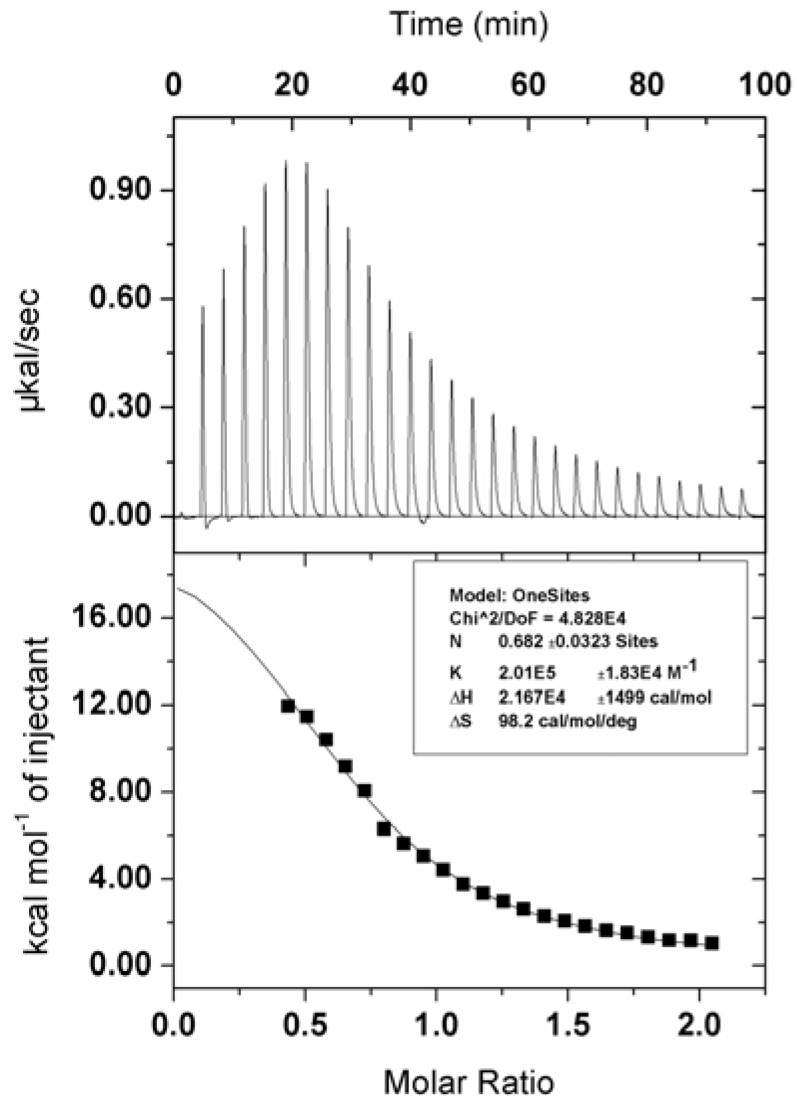
ITC data from the titration of 30 μM BSA in the presence of 300 μM zinc phthalocyanine labelled PEG.

Due to tryptophan resides of BSA, the fluorescence is seen at 348 nm with the exciting wavelength at 280 nm. With the increase of zinc phthalocyanine labelled PEG, the binding to BSA strengthens and the fluorescence of BSA decreases accordingly. The changes in BSA ﬂuorescence intensity are related to the zinc phthalocyanine concentrations by the Stern–Volmer relationship. The Stern–Volmer quenching constant k_SV_ is 3.1623 × 10^5^ and the number of binding sites on BSA(N) in water is 0.85. The N value suggests zinc phthalocyanine labelled PEG forms 1:1 adducts with BSA.The k_SV_ and n are typical of metallated phthalocyanine-BSA interactions in aqueous sloutions,which signifies that phthalocyanine-BSA interactions are not affected after phthalocyanine is conjugated to PEG. In order to clarify the thermodynamic process, ITC has been used to evaluate the binding interaction of zinc phthalocyanine labelled PEG-BSA by quantifying the change in enthalpy entropy and Gibbs free energy as shown in Figure 7 at 25 °C. The raw ITC data is shown at the top, while at the bottom is shown a plot of the heat flow per mole of the titrant *versus* the molar ratio of the titrant to zinc phthalocyanine labelled PEG at each injection. Using computational non-linear fitting analysis, enthalpy change (△H) of 21.67 ± 1.499 kcal mol^−1^ and entropy change (△S) of 98.2 cal in the interaction process indicate that the binding reaction is entropically driven .The binding constant is 2.01 × 10^5^ and the number of binding sites is 0.682,which is in accordance with the data from the fluorescence spectrum.

**Figure 8:**
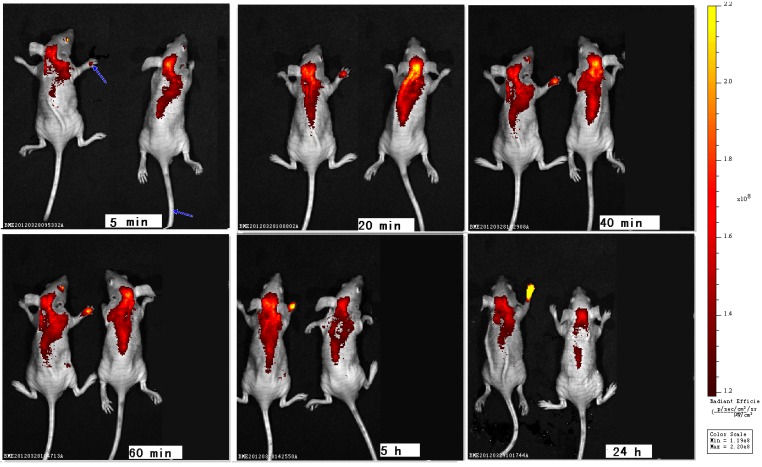
Optical imagings*in vivo* with PEG conjugated zinc phthalocyanines by subcutaneous injection (left) or intravenous injection (right) (blue arrow signifies injection site).

Whole animal imagings were performed in mice to track zinc phthalocyanine labelled PEG from 5 min to 24 h by subcutaneous injection in the right upper paw or intravenous injection in the tail vein (Figure 8). In subcutaneous injection group,weak fluorescence is seen in the paws due to aggregation in 5 min. With the permeation and distribution, the intensity grows from 20 min to 24 h and an obvious distribution and metabolism can be shown.The imaging at 24 h suggests the retention of zinc phthalocyanine labelled PEG in paw with strong fluorescence by subcutaneous injection. In the intravenous injection group, a more rapid distribution and metabolism are seen compared to the subcutaneous injection groupbecause intravenous therapy involves the administration of liquid substances directly into a vein while the rate of distribution of the drug by subcutaneous injection is largely dependent on blood flow and tissue absorption. Compared with other routes of administration, the intravenous route is the fastest way to deliver fluids and medications throughout the body. After 24 h metabolism, the fluorescence mainly concentrates in liver.The change of fluorescence can track and monitor the *in vivo* process of PEG. The results verify the tracking and monitoring effect of zinc phthalocyanine labelled biomaterials.

**Figure 9:**
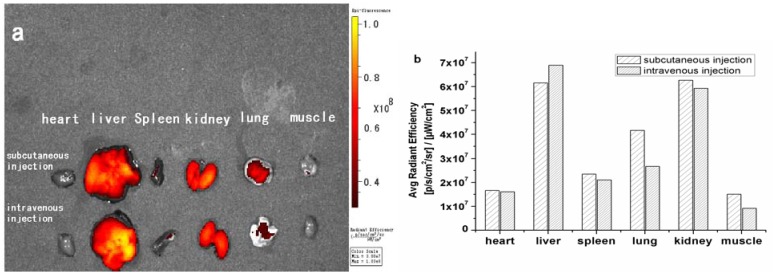
Distribution and fluorescent intensity of dissected organs (**a**), quantitative analysis (**b**).

In order to investigate the organ distribution of PEG conjugated zinc phthalocyanines, the mice were sacriﬁced after 24 h and the organs, including heart, liver, spleen, kidneys, lungs and muscle were harvested for *ex vivo* analysis of material biodistribution. In the subcutaneous injection group and intravenous injection group, strong fluorescence can be seen clearly in the kidney and liver, and secondary fluorescence in the lungs while no fluorescence is observed in heart, spleen and muscle. Quantiﬁcation of ﬂuorescence intensity in whole organs is shown in Figure 9. The organs such as heart, spleen and muscle have only background fluorescence signals. The data reveals that PEG is mainly distributed in kidneys and liver after metabolism by the different injection routes. The two ways of administration have no obvious differences in the distribution of biomaterials after 24 h. 

Safety of biomaterials is a key issue and labeling of biomaterials should cause no damage to tissues or organs. In order to investigate the influence of zinc phthalocyanine labelled PEG on organs, histological analysis was performed. This analysis shows that the zinc phthalocyanine conjugated phthalocyanine does not affect the organs according to the HE staining, which proves the safety of fluorescence label as anear infrared fluorescence agent *in vivo*.

## 3. Experimental

### 3.1. General Methods and Materials

^1^H-NMR spectra were recorded on a Varian Mercury instrument at 300 MHz using CDCl_3_ as solvent and TMS as internal reference. Infrared spectra were recorded on a Nicolet 2000 instrument. UV-Vis and fluorescence spectra were recorded on a Thermo Fisher Scientific Varioskan TM Flash multimode microplate spectra photometer. Isothermal titration calorimetry (ITC) wasmeasured by a VP-ITC calorimeter. Fluorescence images *in vivo* were recorded on a Xenogen IVIS. All purchased materials were used without further purification. 

### 3.2. Synthesis of Zinc Phthalocyanine Labelled PEG

4-Chlorophthalonitrile (**3**, 0.8 g, 5 mmol) and PEG 800 (**2**, 6 g, 7.5 mmol) were mixed in anhydrous DMF(40 mL), then anhydrous K_2_CO_3_ (4 g, 29 mmol) was added. After 24 h mixing at 65 °C, the reaction mixture was ﬁltered, then diluted with dichloromethane and extracted with distilled water. The organic layer was dried over Na_2_SO_4_ and concentrated to get the PEG conjugated phthalonitrile **4**. Without further purification, PEG conjugated phthalonitrile was dissolved in a mixture of DMAE (10 mL) and *n*-butanol (5 mL), then zinc chloride (200 mg, 1.5 mmol) was added. The reaction mixture was stirred under N_2_ for 24 h at 100 °C. After cooling, the solid was reprecipitated by adding cool diethyl ether and collected after filtration to yield a green solid (2.31 g, 48%).

### 3.3. Optical Measurement

The optical characteristics of zinc phthalocyanine labelled PEG were evaluated by UV-Vis and fluorescence spectroscopy in DMSO, water and 5% SDS solution. Quantum yield was measured from the fluorescence spectrum using rhodamine as reference. The concentration dependence of zinc phthalocyanine labelled PEG was evaluated using absorption spectra and fluorescence spectra at concentrations ranging from 1 to 20 μM in DMSO solution. The aggregation behaviors were evaluated using DMSO/water mixed solution at water ratiosof 0%, 25%, 50%, 75% and 100%.

### 3.4. Interaction of Zinc Phthalocyanine Labelled PEG with BSA

The binding of zinc phthalocyanine labelled PEG with BSA was studied by fluorescence spectroﬂuorometry at room temperature. An aqueous solution of BSA (30 μM) was titrated with varying concentrations of the respective zinc phthalocyanine labelled PEG. BSA was excited at 280 nm and ﬂuorescence was recorded between 295 nm and 500 nm. The steady diminution in BSA ﬂuorescence with increase in zinc phthalocyanine concentrations was noted and the changes in BSA ﬂuorescence intensity were related to the concentrations of zinc phthalocyanine by the Stern–Volmer relationship [37]. The thermodynamics of the interaction of zinc phthalocyanine labelled PEG with BSA were measured on a VP-ITC calorimeter. An aqueous solution of BSA (30 μM) was titrated with varying concentrations of the respective zinc phthalocyanine labelled PEG. A typital titration involved 20 injections of zinc phthalocyanine labelled PEG interacted in the sample cell. The titration cell was stirred continuously at 310 rpm. The data were analyzed to determine the binding stoichiometry (N), and affinity constant (K) using the Origin software.

### 3.5. *In Vivo* Imaging and Distribution of Zinc Phthalocyanine Labelled PEG

Athymic nude mice (seven weeks old, 20–25 g) were used. All the animal experiments were performed in compliance with the Guiding Principles for the Care and Use of Laboratory Animals, Peking Union Medical College, China. Animals had free access to food and water. Athymic nude mice were randomly assigned to different groupsas follows: subcutaneous injection group, intravenous injection, control group (n = 3 for each group). In the experimental group, zinc phthalocyanine labelled PEG solution was administered by subcutaneous injection in the right upper paw or by intra venous injection in the tail vein at 100 μL of 200 μM. Images were taken using a Xenogen IVIS (filters: excitation 640 nm, emission 705 nm) with an exposure time of 4 s after 5 min, 20 min, 40 min, 60 min, 5 h and 24 h respectively. At the end of the imaging, anesthetized mice were sacrificed and images of organs were made to evaluate the distribution of near infrared fluorescence agent. Fluorescence images of organs were analyzed using the Xenogen Analysis Software. After imaging, organ tissues were immediately immersed into 4% formaldehyde in phosphate-buffered saline of pH 7.4 at 4 °C for 24 h. After fixation, the samples were embedded in paraffin and sectioned to 5-μm-thick slices. Routine staining was performed with hematoxylin-eosin.

## 4. Conclusions

In summary, zinc phthalocyanine labelled PEG has been prepared and evaluated. The fluorescence labelled biomaterials have beneficial optical stability and fluorescence quantum yield and moderate interations with BSA. Near-infrared imaging effect, distribution in organs as well as histological analysis demonstrate that zinc phthalocyanine labelling can be used as a real-time noninvasive method for tracking and monitoring of PEG materials *in vivo* with good biocompatibility. The results show the potential of this technique to develop a new class of fluorescence biomaterials that could allow the monitoring of *in vivo* processes and distribution of biomaterial implants or drug carriers in patients.
